# Near-Complete Genome Sequence of Zygosaccharomyces rouxii NRRL Y-64007, a Yeast Capable of Growing on Lignocellulosic Hydrolysates

**DOI:** 10.1128/mra.00050-22

**Published:** 2022-04-20

**Authors:** Sujit Sadashiv Jagtap, Jing-Jing Liu, Hanna E. Walukiewicz, Jasmyn Pangilinan, Anna Lipzen, Steven Ahrendt, Maxim Koriabine, Kelly Cobaugh, Asaf Salamov, Yuko Yoshinaga, Vivian Ng, Chris Daum, Igor V. Grigoriev, Patricia J. Slininger, Bruce S. Dien, Yong-Su Jin, Christopher V. Rao

**Affiliations:** a Department of Chemical and Biomolecular Engineering, University of Illinois at Urbana-Champaign, Urbana, Illinois, USA; b DOE Center for Advanced Bioenergy and Bioproducts Innovation, University of Illinois at Urbana-Champaign, Urbana, Illinois, USA; c U.S. Department of Energy Joint Genome Institute, Lawrence Berkeley National Laboratory, Berkeley, California, USA; d Department of Plant and Microbial Biology, University of California, Berkeley, Berkeley, California, USA; e Bioenergy Research Unit, National Center for Agricultural Utilization Research, USDA-Agricultural Research Service, Peoria, Illinois, USA; f Department of Food Science and Nutrition, University of Illinois at Urbana-Champaign, Urbana, Illinois, USA; University of California, Riverside

## Abstract

The halotolerant and osmotolerant yeast Zygosaccharomyces rouxii can produce multiple volatile compounds and has the ability to grow on lignocellulosic hydrolysates. We report the annotated genome sequence of Z. rouxii NRRL Y-64007 to support its development as a platform organism for biofuel and bioproduct production.

## ANNOUNCEMENT

Zygosaccharomyces rouxii NRRL Y-64007 is a halotolerant, osmotolerant, acidophilic, and fructophilic yeast (1–3). It is used for the production of volatile compounds, organic acids, lipids, and sugar alcohols (4–10). This yeast was initially called Lipomyces starkeyi Y-11557 but was renamed Z. rouxii NRRL Y-64007 based on the internal transcribed spacer (ITS) sequence (GenBank accession number OM905798) and genome sequencing results (9). In this study, we sequenced the genome and transcriptome of Z. rouxii NRRL Y-64007 to facilitate further investigation of its physiology, metabolism, and metabolic engineering to produce biofuels and bioproducts.

YPG medium (10 g/L yeast extract, 20 g/L peptone, and 20 g/L glucose) was used for the growth of Z. rouxii NRRL Y-64007. Cell cultures with a total optical density at 600 nm (OD_600_) of 10 were used for genomic DNA and RNA extraction. The genomic DNA and total RNA of Z. rouxii NRRL Y-64007 were extracted using the Dr. GenTLE (from yeast) high recovery kit (TaKaRa Bio Inc., Shiga, Japan) and the RNeasy minikit (Qiagen, Hilden, Germany), respectively (11, 12).

For genome sequencing, libraries of >10 kb were prepared for Pacific Biosciences (PacBio) sequencing using 5 μg of genomic DNA as reported previously and according to the PacBio template preparation and sequencing guide (13, 14). The sheared DNA was treated with exonuclease, followed by end repair and ligation of blunt adapters using the SMRTbell template preparation kit v1.0. The library was purified with AMPure PB beads with a 10-kb cutoff value. The prepared SMRTbell template libraries were then sequenced on a PacBio Sequel II sequencer using v3 sequencing primer, 8M v1 single-molecule real-time (SMRT) cells, and v2.0 sequencing chemistry with 1 × 900-bp sequencing movie run times. BBduk and BBMerge from BBTools v36.63 (https://sourceforge.net/projects/bbmap) were used to remove reads aligning to human, cat, dog, mouse, and common microbial contaminants (15).

Plate-based RNA sample preparation was performed with the PerkinElmer Sciclone next-generation sequencing (NGS) robotic liquid handling system using the Illumina TruSeq stranded mRNA high-throughput (HT) sample preparation kit with poly(A) selection of mRNA, following the protocol outlined by Illumina in the user guide (16); 1 μg RNA per sample and eight cycles of PCR were used for library amplification. The prepared libraries were quantified using the KAPA Biosystems NGS library quantitative PCR (qPCR) kit and run on a Roche LightCycler 480 real-time PCR instrument. Sequencing was performed using NovaSeq XP v1 reagent kits and an S4 flow cell. An Illumina library was constructed and sequenced (2 × 151 bp) using the Illumina NovaSeq S4 system. BBDuk v38.79 (http://bbtools.jgi.doe.gov) was used to remove contaminants, to trim reads that contained adapter sequences, and to quality trim reads with quality scores of <6. Reads mapped with BBMap to common contaminants and rRNA reads were removed. Filtered fastq files were used as input for *de novo* assembly of RNA contigs. Reads filtered and trimmed for quality and contamination were assembled into consensus sequences using Trinity v2.8.5 (17).

The 9.95-Mb genome assembly contained 8 contigs (*N*_50_, 1.53 Mb), with sequencing read coverage depth of 181.58× and a GC content of 39.12%. The genome was annotated using the JGI annotation pipeline (18, 19) to predict 5,001 protein-coding genes. A noncanonical telomere consensus sequence was checked to assess the near-complete genome; it was present at both the start and the end of the first 4 scaffolds and only at the end of scaffolds 5, 6, 7, and 8.

### Data availability.

The whole-genome assembly and annotation are available from MycoCosm (https://mycocosm.jgi.doe.gov/Zygrou1) (17). This whole-genome shotgun project has been deposited in DDBJ/ENA/GenBank under the accession number JAKETS000000000. The version described in this paper is JAKETS010000000. The accession numbers for the BioProject and reads are PRJNA784295 and SRR17438072, respectively.
